# Characterization of the Tibet plateau Jerusalem artichoke (*Helianthus tuberosus* L.) transcriptome by de novo assembly to discover genes associated with fructan synthesis and SSR analysis

**DOI:** 10.1186/s41065-019-0086-8

**Published:** 2019-02-06

**Authors:** Shipeng Yang, Xuemei Sun, Xiaoting Jiang, Lihui Wang, Jie Tian, Li Li, Mengliang Zhao, Qiwen Zhong

**Affiliations:** 1 Academy of Agriculture and Forestry Sciences of Qinghai University (Qinghai Academy of Agriculture and Forestry Sciences), Qinghai Key Laboratory of Vegetable Genetics and Physiology, State Key Laboratory of Plateau Ecology and Agriculture, Qinghai University, Xining, 810016 China; 2 Qinghai Higher Vocational & Technical College Institute, Ledu, 810799 China

**Keywords:** *Helianthus tuberosus* L., Transcriptome, Gene analysis, Simple sequence repeats

## Abstract

**Background:**

Jerusalem artichoke (*Helianthus tuberosus* L.) is a characteristic crop in the Qinghai-Tibet Plateau which has rapidly developed and gained socioeconomic importance in recent years. Fructans are abundant in tubers and represent the foundation for their formation, processing and utilization of yield; and are also widely used in new sugar-based materials, bioenergy processing, ecological management, and functional feed. To identify key genes in the metabolic pathway of fructans in Jerusalem artichoke, high-throughput sequencing was performed using Illumina Hi Seq™ 2500 equipment to construct a transcriptome library.

**Results:**

Qinghai-Tibet Plateau Jerusalem artichoke “Qingyu No.1” was used as the material; roots, stems, leaves, flowers and tubers of Jerusalem artichoke in its flowering stage were mixed into a mosaic of the Jerusalem artichoke transcriptome library, obtaining 63,089 unigenes with an average length of 713.6 bp. Gene annotation through the Nr, Swiss Prot, GO, KOG and KEGG databases revealed 34.95 and 46.91% of these unigenes had similar sequences in the Nr and Swiss Prot databases. The GO classification showed the Jerusalem artichoke unigenes were divided into three ontologies, with a total of 49 functional groups encompassing biological processes, cellular components, and molecular functions. Among them, there were more unigenes involved in the functional groups for cellular processes, metabolic processes, and single-organism processes. 38,999 unigenes were annotated by KOG and divided into 25 categories according to their functions; the most common annotation being general function prediction. A total of 13,878 unigenes (22%) were annotated in the KEGG database, with the largest proportion corresponding to pathways related to carbohydrate metabolism. A total of 12 unigenes were involved in the synthesis and degradation of fructan. Cluster analysis revealed the candidate 12 unigene proteins were dispersed in the 5 major families of proteins involved in fructan synthesis and degradation. The synergistic effect of INV gene is necessary during fructose synthesis and degradation in Jerusalem artichoke tuber development. The sequencing data from the transcriptome of this species can provide a reliable data basis for the identification and assessment of the expression of the members of the INV gene family.A simple sequence repeat (SSR) loci search was performed on the transcriptome data of Jerusalem artichoke, identifying 6635 eligible SSR loci with a large proportion of dinucleotide and trinucleotide repeats, and the most different motifs were repeated 5 times and 6 times. Dinucleotide and trinucleotide repeat motifs were the most frequent, with AG/CT and ACC/GGT repeat motifs accounting for the highest proportion.

**Conclusions:**

In this study, a database search of the transcriptome of the Jerusalem artichoke from the Qinghai Tibet Plateau was conducted by high throughput sequencing technology to obtain important transcriptional and SSR loci information. This allowed characterization of the overall expression features of the Jerusalem artichoke transcriptome, identifying the key genes involved in metabolism in this species. In turn, this offers a foundation for further research on the regulatory mechanisms of fructan metabolism in Jerusalem artichoke.

## Background

Jerusalem artichoke (*Helianthus tuberosus* L.) belongs to the genus *Helianthus L*. of Asteraceae. Although it is native to temperate zones in North America, it was introduced into China over 300 years ago, where it has been planted sporadically as a pickled vegetable for a long time. The twenty-first Century has seen a gradual decrease of the economic value of Jerusalem artichoke, with a rapid expansion in its planting and processing. The Jerusalem artichoke tuber contains fructose, which can be used to process inulin [1], bioethanol [2] and various foods [3]. In addition, because of its strong stress resistance, it can be cultivated in dry and saline alkaline environments, and has also been utilized in the ecological treatment of desert soil [4], seashore saline soil [5] and heavy metal-contaminated soil [6]. The Qinghai-Tibet Plateau is one of the earliest areas in China to conduct large-scale planting and processing of Jerusalem artichoke. The climate is cold, with large differences in temperature between day and night. The yield of the Jerusalem artichoke tuber is high, due its elevated fructan content. Moreover, it has natural and pollution-free environmental advantages, being very beneficial to the processing of organic fructan products. The industry of Jerusalem artichoke planting and processing has achieved rich development potential in the Qinghai-Tibet Plateau.

Fructose is the third largest storage carbohydrate after starch and sucrose. About 15% of angiosperms contain fructan, most prominently in the Compositae, Liliaceae and Gramineae families; which mostly grow in cold temperate zones [7]. Plant fructan has been studied for over 200 years, with early research focused on the biochemical properties of fructans [8]. In recent years, abundant studies have shown fructan metabolism is closely related to plant resistance to cold [9], drought [10] and saline alkali [11], as well as other stressors. With the discovery of the enzymes involved in fructan metabolism, research focus has shifted to the understanding of the metabolic processes and functions of fructosan [12]. In addition to the important role of fructan in plants, processed food derived from this carbohydrate also helps maintain human health. The properties and processing techniques of fructan have been extensively studied [13]. Fructan is an optimal nutrient and sweetener for patients with diabetes and hypertension, as it has a caloric value of only 1 kcal/g, and it is not easily digested and decomposed into monosaccharides and therefore does not sharply increase blood glucose [14, 15]. Highly polymerized fructans are often used as substitutes for fats in foods such as yogurt, ice cream and jelly pudding [16].

Fructan structure is mainly linear or branched in plants and 5 different types of derivatives. The earliest model of fructan metabolism was proposed by research on Jerusalem artichoke [17]. Jerusalem artichoke is a typical crop that accumulates inulin-type linear fructan. This type is composed of D-fructose residues linked by β-2, 1 bonds with molecular weights ranging from 3 to 5 kDa [18]. Current models of inulin-type fructan metabolism suggest its synthesis is based on sucrose as a substrate: First, the synthesis of kestose (1-Kestose) is catalyzed by sucrose 1-fructosyltransferase (1-SST, EC 2.4.1.99), followed by elongation of the fructan chain [19, 20] catalyzed by fructan 1-fructosyltransferase (1-FFT, EC 2.4.1.100). Then, the degradation of fructan is catalyzed by 1-Fructan exohydrolase (1-FEH, EC 3.2.1.80), with the fructosyl being cleaved one by one from the end of the sugar chain. At present, the functions of known key enzymes in the metabolism of Jerusalem artichoke fructan are unclear, and which genes are involved in their metabolic regulation remains to be discovered.

In recent years, high-throughput transcriptome sequencing has been widely used to study gene expression in different biological problems. This method can comprehensively and quickly acquire all mRNA sequences [21–23] generated by gene expression in a certain state and identify important functional genes from it in order to reveal the molecular mechanisms underlying different biological traits. In this study, the transcriptome of Jerusalem artichoke from the Qinghai Tibet Plateau was sequenced using high-throughput sequencing technology with Illumina Hi Seq 2500 equipment. Functional annotation of genes, and functional classification and metabolic pathway analysis of unigene were carried out with bioinformatics methods to lay a foundation for further research on the key metabolic genes in Jerusalem artichoke, as well as for interpretation of the molecular mechanisms involved in the regulation of fructan metabolism, and the development of related molecular markers.

## Results

### Analysis of RNA-Seq datasets

**Fig. 1:**
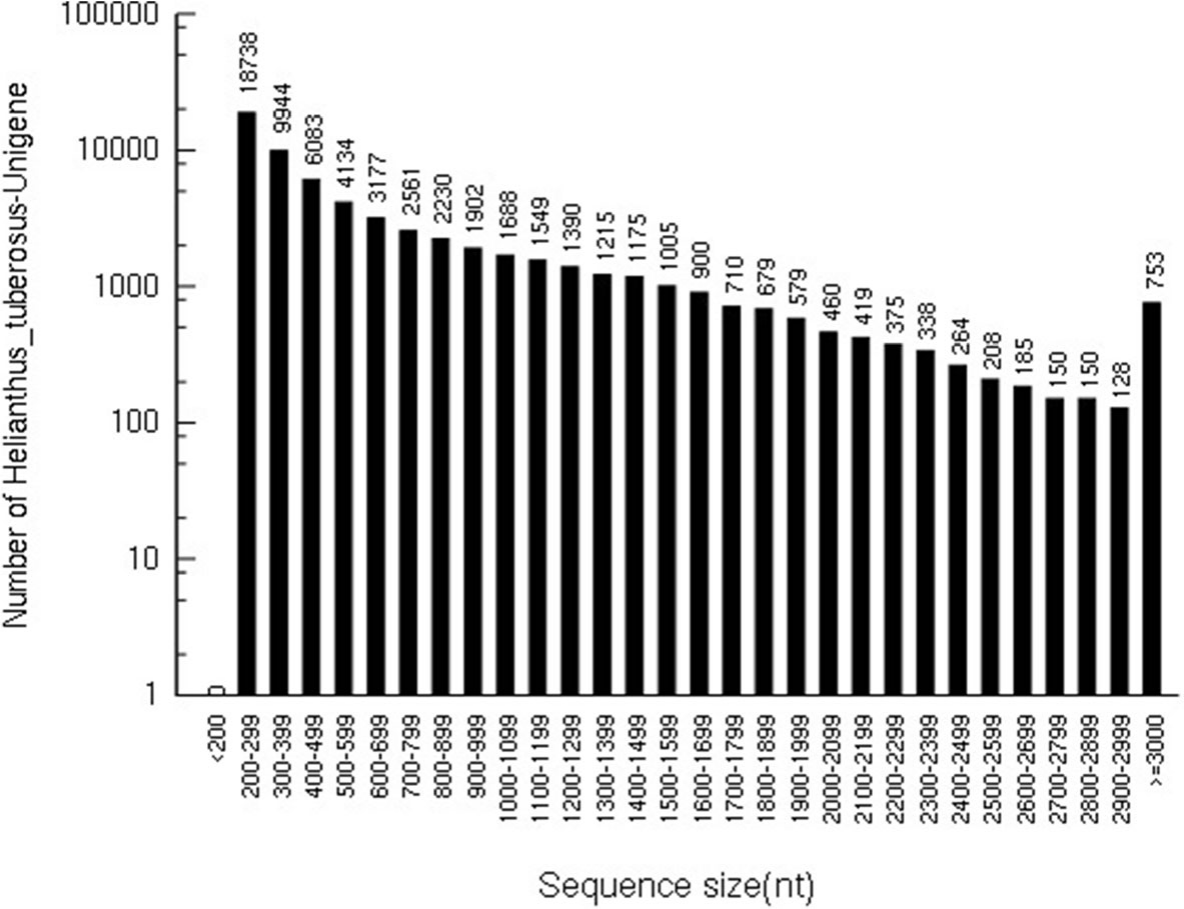
Size range of unigenes from transcriptome data of Jerusalem artichoke

Illumina sequencing obtained a total of 123,169,632 reads, containing 18.68 G of nucleotide sequence information. The percentages of Q20 (sequencing error rate less than 1%), Q30 (sequencing error rate less than 0.1%), and GC content were 98.28, 95.40, and 44.92%, respectively (Table 1). The data from the transcriptome sequencing had high volume and quality, providing good raw data for subsequent data assembly. Trinity was assembled to obtain 63,098 spliced transcript sequences, with an average length of 713 nt and a N50 of 1106 nt. The longest transcript in each gene was taken as an unigene, with an average length of 713.6 bp. The length distribution of the unigenes revealed a total of 12,962 unigenes with a length greater than 1000 nt, accounting for 20.55% of all unigenes. Among them, 34,765 unigenes had a length of 200–499 nt, accounting for 55.10% of the total; whereas 14,004 unigenes had a length of 500–999 nt, corresponding to 22.20% of the total; and 14,320 unigenes had a length of ≥1000 nt, accounting for 22.70% of the total (Fig. 1). This indicated the transcriptome library had good sequencing and assembly results, and adequate for subsequent bioinformatics analysis.

**Table 1:** Transcriptome Sequence data quality for the Jerusalem artichoke

Raw reads	Clean reads	Clean bases	Q20(%)^a^	Q30(%)^b^	GC(%)
124,500,360	123,169,632	18.68G	98.28%	95.40%	44.92%

^a^Q20: percentage is the proportion of nucleotides with a quality > 20 in reads

^b^Q30: percentage is the proportion of nucleotides with a quality > 30 in reads

### Functional annotation and classification

The assembled unigene sequences were separately aligned according to the Nr and Swissprot databases by Blast software. Results recognized 22,048 unigenes aligned to similar sequences in the Nr database, with E values ranging from 1E-50 to 1E-20, with a total of 9873 (44.78%). To determine the species to which homologous sequences belong, the sequence in which each unigene yielded the best results (the lowest E value) in the Nr library was selected (if there was a parallel, the first one was selected). The number of homologous sequences statistically aligned to each species was determined in the Nr database. The highest matches with the unigenes from Jerusalem artichoke were *Cynara cardunculus* (19002), followed by *Daucus carota* (1346), *Helianthus annuus* (1126), *Vitis vinifera* (1042), *Theobroma cacao* (934), *Sesamum indicum* (810), and others. 29,595 (46.91%) unigenes had similar sequences in the Swiss Prot database, where the E values of most ranged between 1E-50 to 1E-20, corresponding to 8135 unigenes (27.49%). Compared with Nr, SwissProt function annotation found a large reduction in unigenea with high similarity sequences, consistent with database characteristics. The SwissProt data source is very strict, and all sequence entries are carefully checked by experienced molecular biologists and protein chemists through computer tools and related literature. In contrast, the Nr database integration standard is looser, and any two sequences any differences are regarded as two distinct records.

**Fig. 2:**
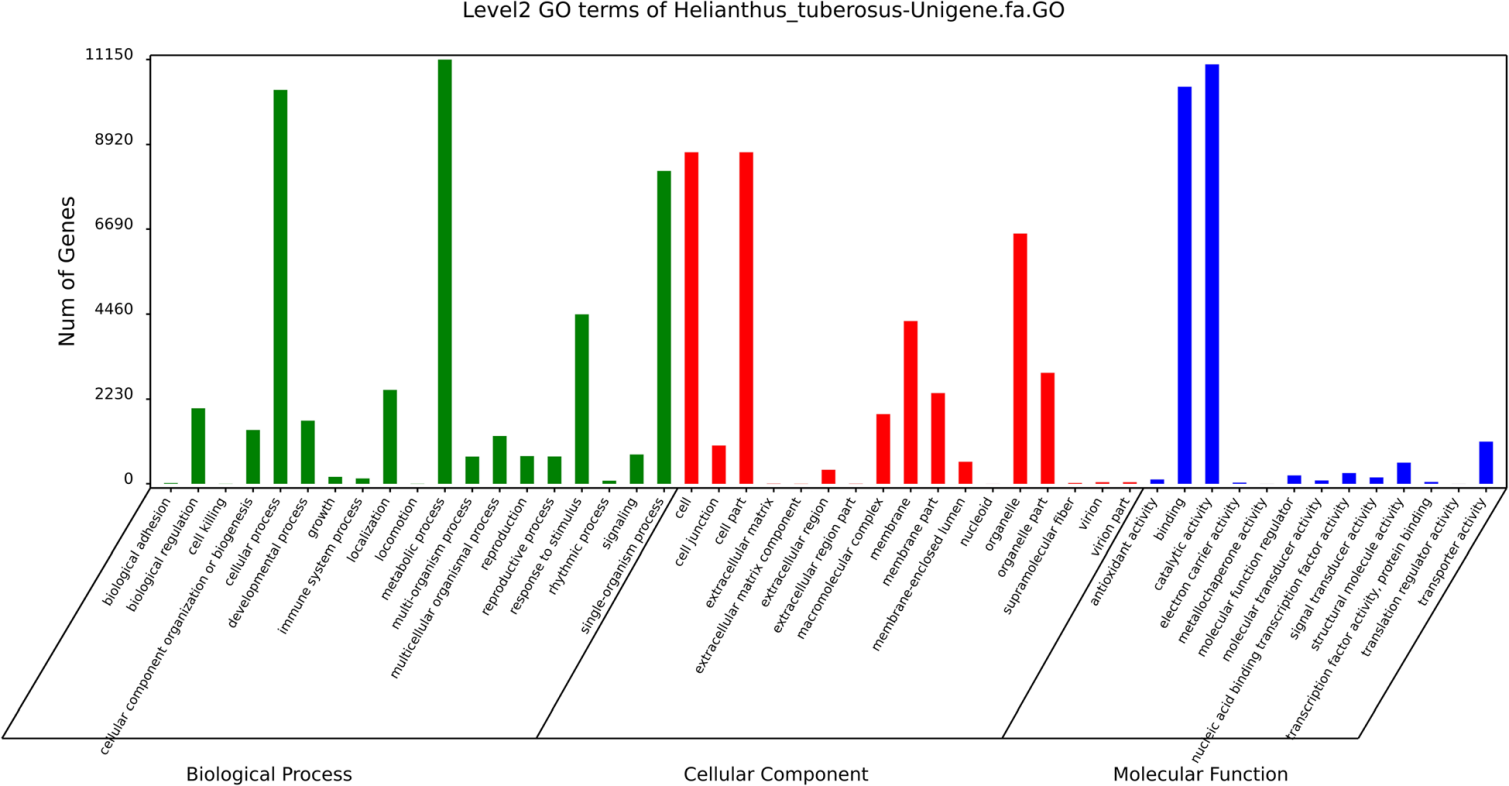
Gene ontology (GO) classification of assembled unigenes in Jerusalem artichoke

The GO database was used to classify the unigenes according to biologic function. Results showed the 63,089 Jerusalem artichoke unigenes were divided into three ontologies (Fig. 2): Biological processes, Cellular components, and Molecular functions, with a total of 49 functional groups. Further analysis showed 46,360 GO entries belonged to 19 functional groups related to biological processes; 24,082 corresponded to 17 functional groups linked to cellular components; and 374,83 GO entries belonged to 13 functional groups related to molecular functions. Most unigenes were allocated to the functional groups of cellular processes (10,358 unigenes), metabolic processes (11,147 unigenes), the single-organism processes (8223 unigenes), the cell (8716 unigenes), cell parts (8716 unigenes), binding (10,443); while the lowest amount of unigenes were involved in the functional groups of cell killing (3 unigenes), locomotion (4 unigenes), metallochaperone activity (1 unigenes), translation regulator activity (1 unigenes), nucleoid (1 unigenes), extracellular matrix components (4 unigenes), extracellular region parts (5 unigenes) and the extracellular matrix (6 unigenes).

**Fig. 3:**
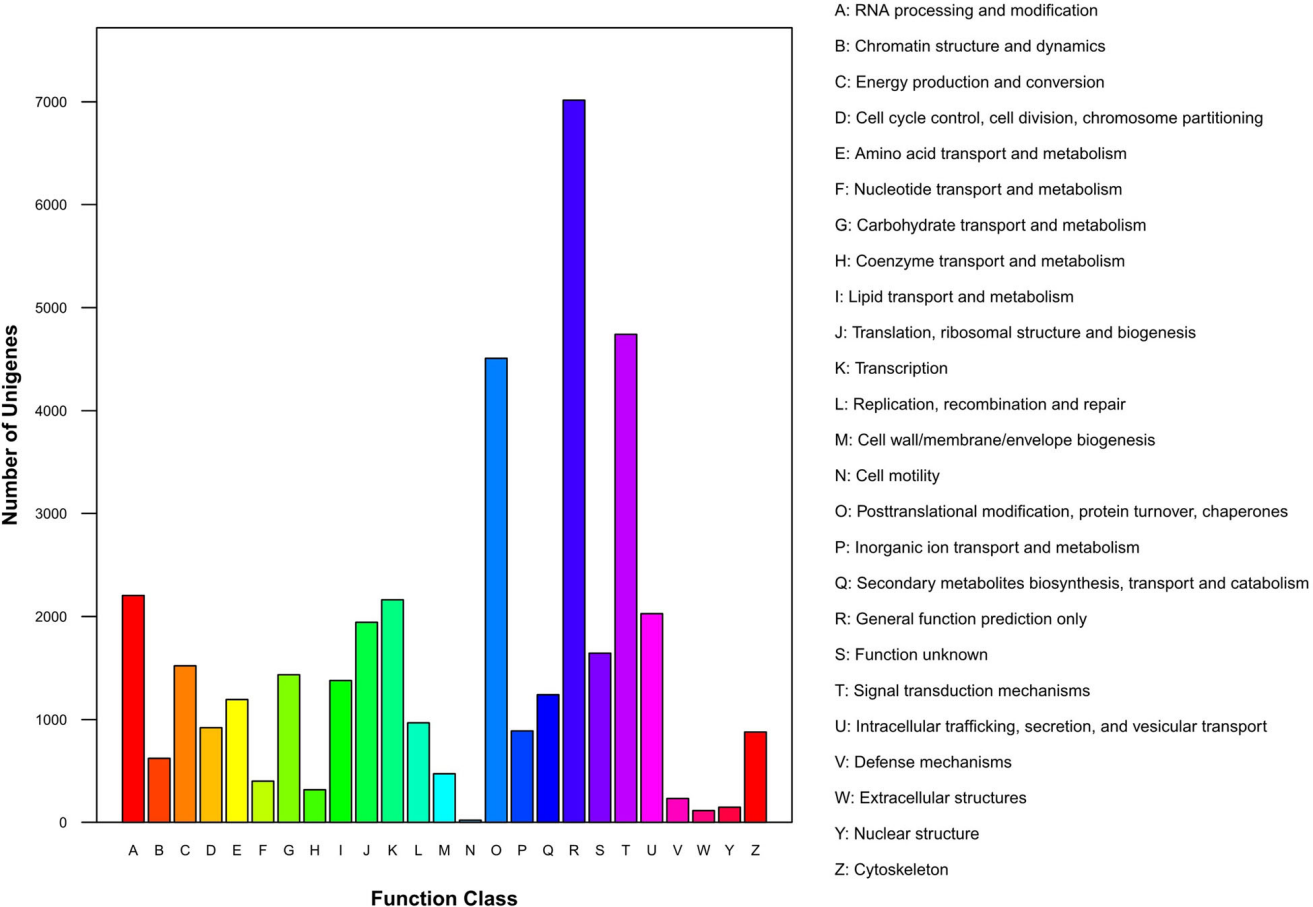
Clusters of orthologous groups for eukaryotic complete genomes (KOG) classification of assembled unigenes

Jerusalem artichoke unigenes were compared with the KOG database and classified according to their function. 38,999 unigenes were divided into 25 categories, with the most annotated unigenes corresponding to General function prediction (7016 unigenes), followed by those related to posttranslational modification, protein turnover and chaperones (4507 unigenes), and signal transduction mechanisms (4740 unigenes). Meanwhile, the groups with the least annotated unigenes were those relate to nuclear structure (147 unigenes), extracellular structures (114 unigenes), and cell motility (21 unigenes). The identified unigenes of Jerusalem artichoke were involved in most biological activities typical of growth and development (Fig. 3).

### Functional classification by the KEGG pathway

**Fig. 4:**
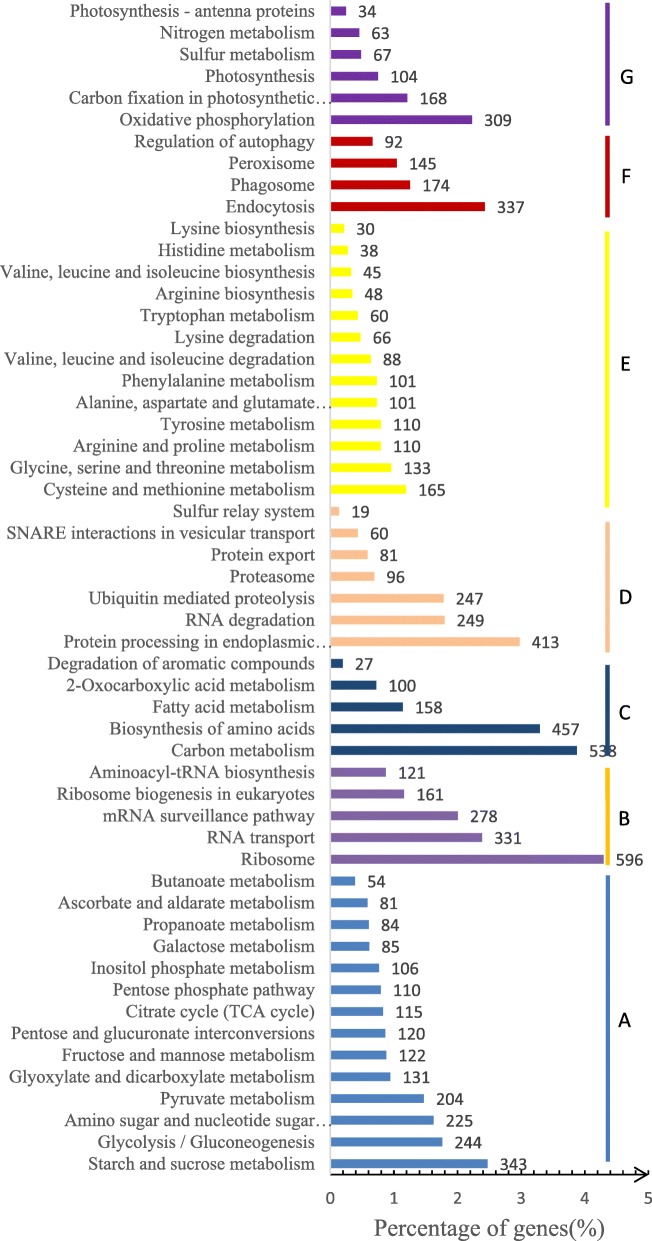
KEGG analysis of Jerusalem artichoke unigenes for metabolic pathways. A: Carbohydrate metabolism. B: Translation. C: Global and Overview. D: Folding, sorting and degradation. E: Amino acid metabolism. F: Transport and catabolism. G: Energy metabolism

Jerusalem artichoke unigenes were compared to the KEGG database, and the metabolic pathways were analyzed based on the annotation information to understand the metabolic pathways and functions of their products in cells. A total of 13,878 unigenes (22.00%) were annotated for analysis of metabolic pathways. Among the 7 most statistically significant pathways, carbohydrate metabolism-related pathways accounted for the largest proportion, followed by translation, global and overview, folding, sorting and degradation, amino acid metabolism, transport and catabolism, and energy metabolism. Most unigenes corresponded to metabolic pathways, indicating Jerusalem artichoke had strong metabolism during this period. On the other hand, more than 400 other unigenes were linked to other subclasses related to ribosomes, carbon metabolism, biosynthesis of amino acids, protein processing in the endoplasmic reticulum, and plant hormone signal transduction pathways (Fig. 4).

### Phylogenetic analysis of fructan synthesis and degradation unigenes

**Fig. 5:**
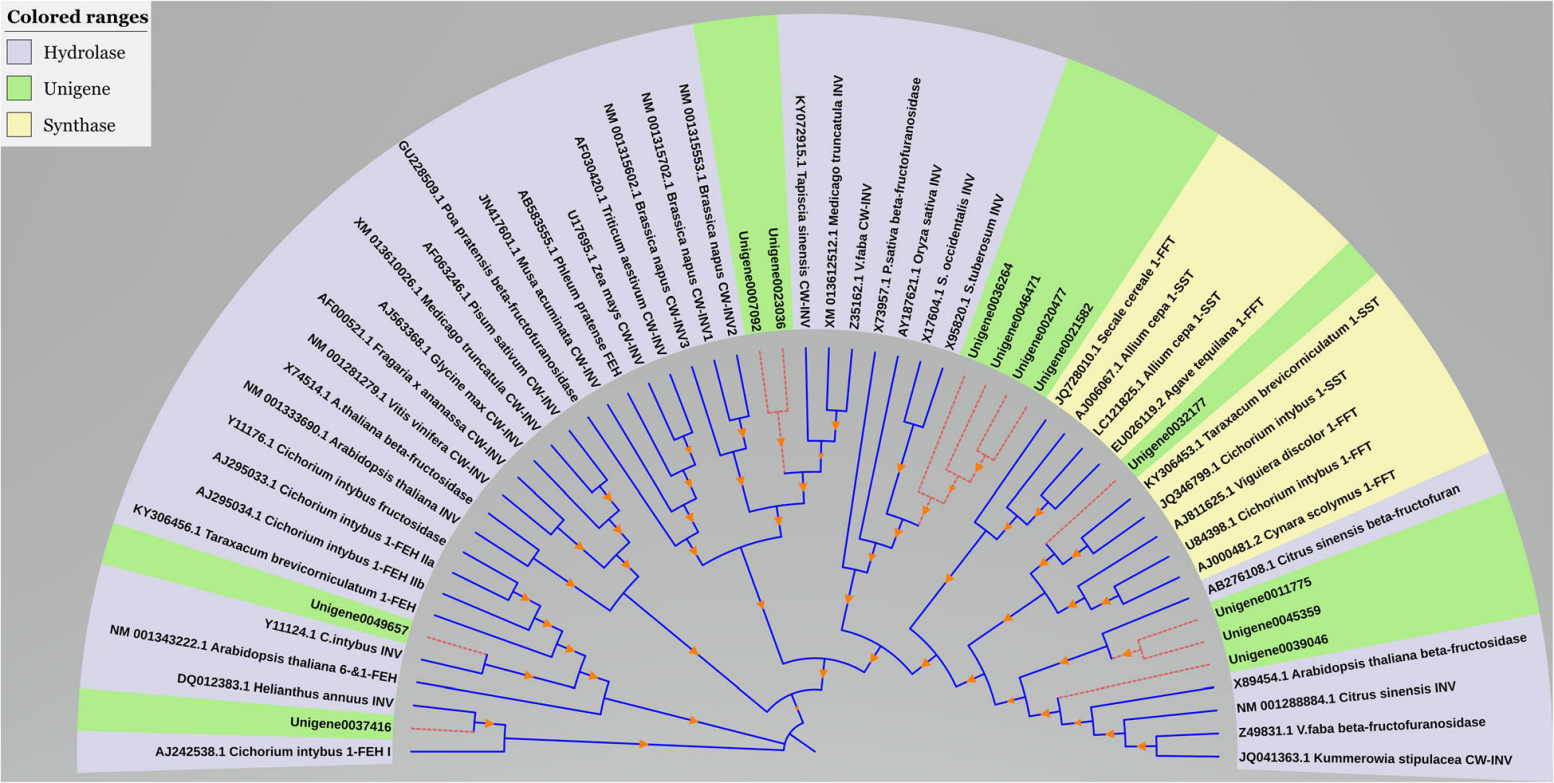
Phylogenetic analysis of 32 families of glycosyl hydrolase from Jerusalem artichoke and different species

Results indicated 12 unigenes were involved in the synthesis and degradation of fructan (Table 2). The protein phylogenetic tree of Jerusalem artichoke and other fructan crops was constructed by MEGA 5.0 software. According to the topology of the phylogenetic tree (Fig. 5), fructan synthesis and degrading enzyme gene proteins were clustered and analyzed into three categories: nigenes, fructosyltransferase gene and fructosyl synthase gene, obtained by sequencing. The 12 candidate unigenes’ proteins were dispersed in five major protein families of fructan synthesis and degradation enzymes; especially in the fructosyltransferase gene family, accounting for 91.67%. This may be because the fructosyltransferase gene is similar in structure to the vacuolar invertase and cell wall invertase genes, displaying the widest distribution in the phylogenetic tree. The fructosyltransferase gene were clustered with Unigene37416, Unigene49657, Unigene7092 and Unigene23036. These fructosyltransferase genes awere basically INV, CW-INV and β-fructofuranosidase. However, Unigene32177 was the only candidate unigene that was combined with the fructan synthase protein. In addition, Unigene37416 and Unigene49657 were on the same evolutionary branch as *Helianthus annuus* and *Cichorium intybus*, respectively. Unigene36264, Unigene46471, Unigene20477, and Unigene21582 were adjacent and on the same sub-branches, and Unigene11775, Unigene45359, and Unigene39046 shared the same distribution, suggesting they may have similar functions.

**Table 2:** Information of candidate 12 fructan pathways genes in the transcriptome of Jerusalem artichoke

Gene ID	Length(bp)	Blast result		
		Species	E value	Annotation
Unigene0007092	1084	*Solanum tuberosum*	5.00E-162	Apoplastic invertase
Unigene0011775	1982	*Solanum tuberosum*	2.00E-60	Vacuolar invertase
Unigene0020477	862	*Daucus carota*	1.00E-123	Cell wall beta-fructosidase(Inv1)
Unigene0021582	445	*Rhus chinensis*	4.00E-62	Beta-fructofuranosidase
Unigene0023036	726	*Populus grandidentata*	9.00E-116	Cell-wall invertase
Unigene0032177	2094	*Cichorium intybus*	3.00E-32	Sucrose:sucrose 1-fructosyltransferase
Unigene0036264	1987	*Cichorium intybus*	1.00E-10	Beta-fructofuranosidase
Unigene0037416	1838	*Helianthus annuus*	6.00E-36	Cell wall invertase (Cw-inv1)
Unigene0039046	2047	*Cynara cardunculus*	1.00E-126	Cell wall beta-fructosidase(Inv2)
Unigene0045359	2175	*Lycium barbarum*	2.00E-25	Acid invertase
Unigene0046471	1841	*Cichorium intybus*	2.00E-11	Invertase
Unigene0049657	1709	*Manihot esculenta*	4.00E-62	Cell wall invertase

### Candidate genes involved in fructan metabolism and expression analysis of 12 candidate genes by quantitative polymerase chain reaction

**Fig. 6:**
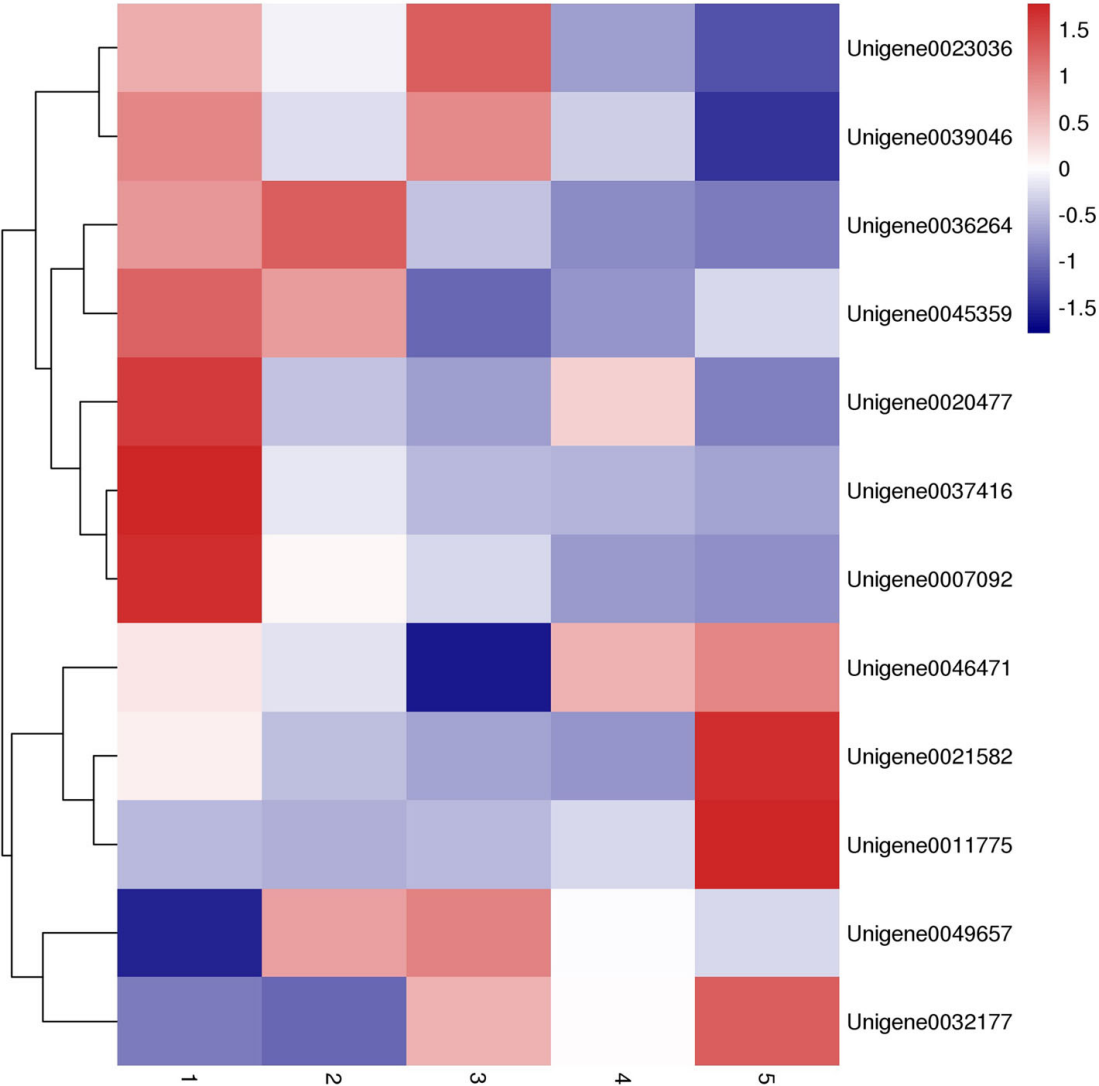
Quantitative analysis of gene expression during the formation of Jerusalem artichoke tubers: 1(Date: 7.15). 2(Date: 8.1). 3(Date: 8.15). 4(Date: 9.1). 5(Date: 9.15)

Assessment with quantitative polymerase chain reaction (qPCR) was performed in Jerusalem artichoke samples at the tuber formation stage to evaluate the expression of 12 unigenes related to the fructan metabolism. Changes in the expression of Cell wall-INV related Unigene23036 and Unigene39046 were similar Jerusalem artichoke had increased height and tubers had matured. The highest value was reached in the third stage and then gradually decreased, showing fluctuation (Fig. 6). The decomposition of sucrose is mainly catalyzed by INV in higher plants. INV is a key enzyme gene regulating sucrose degradation in Jerusalem artichoke, and can hydrolyze sucrose to glucose and fructose, which are the substrates for fructan synthesis. Interestingly, the INV-related Unigene36264, Unigene45359, Unigene20477, Unigene37416 and Unigene07092 were expressed at higher levels in the early stage of tuber development. With gradual tuber expansion, expression gradually decreased, with a slight drop after the initial second stage of tuber expansion, and remaining relatively stable in the late stage of tuber development. This difference in expression patterns indicates each type of INV may have different biological functions. In addition, Unigene21582, Unigene11775, Unigene49657 and Unigene32177 showed up-regulated expression throughout the whole development and expansion process of the Jerusalem artichoke tuber. The expression level continued to rise, especially from September 1st to the harvest season,. These unigenes may be associated with fructan accumulation in the late stage of tuber development of this species. The distinct expression characteristics of these INV-related unigenes indicate there is functional differentiation during the evolution process. In general, these unigenes are highly expressed in the tuber development stage; suggesting they may play an important role in the synthesis and degradation of fructan. However, further research is required to elucidate specific molecular mechanisms.

### Expressed sequence tag-simple sequence repeat discovery: distribution and frequencies

A total of 63,089 unigenes (with a total length of about 45,020,635 bp) were searched in the transcriptome of Jerusalem artichoke with MISA software, identifying 6635 eligible SSR loci in 5452 unigenes, with a 10.52% occurrence frequency (SSR locus/total unigene number) and 8.64% incidence frequency (the number of unigenes containing the SSR loci/the total number of unigenes). Among these unigenes containing SSR loci, 4521 (82.92%) contained 1 SSR locus, whereas the remaining 931 (17.08%) contained ≥2 SSR loci. Regarding distribution frequency, the ratio of dinucleotides and trinucleotides was relatively large, accounting for 45.24 and 42.53% of the total, respectively. Meanwhile, concerning the repetition of different primitives, the frequency of 4–9 times repeats was higher, accounting for 95.31% of the total. Among them, they were mainly 5- and 6-time repeats, which corresponded to 1887 and 2059 unigenes, respectively, and accounted for 28.44 and 31.03% of the total (Table 3).

**Table 3:** Distribution of SSR types in the transcriptome of *Helianthus tuberosus* L

Repeat number	Motif type					Total	Ratio (%)
	Di-	Tri-	Tetra-	Penta-	Hexa-		
4	0	0	360	135	111	606	9.13
5	0	1736	92	27	32	1887	28.44
6	1301	732	22	2	2	2059	31.03
7	733	247	10	3	2	995	14.99
8	456	41	2	2	3	504	7.59
9	256	16	0	0	1	273	4.11
> 9	256	50	3	0	2	311	4.69
Total	3002	2822	489	169	153	6635	
Proportion%	45.24	42.53	7.37	2.55	2.31		100.00

**Fig. 7:**
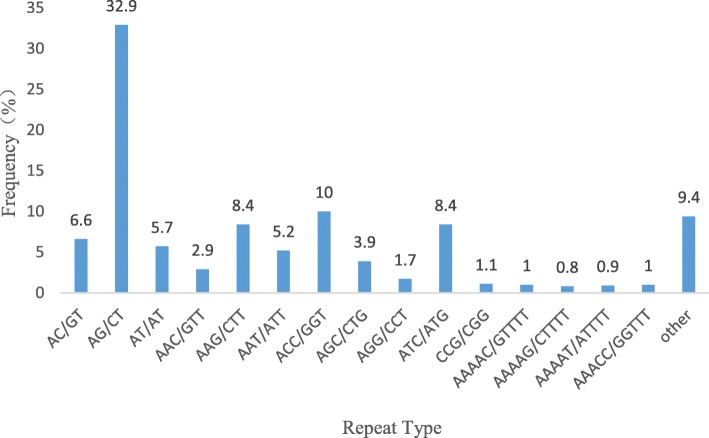
Proportions of SSR among the total tandem repeats in Jerusalem artichoke

We found the Jerusalem artichoke transcriptome to be rich in SSR types, with 6635 SSR loci containing 180 repeat motifs, corresponding to 4 dinucleotides, 10 trinucleotides, 30 tetranucleotides, 52 petanucleotides and 83 hexanucleotides. Among the dinucleotides, AG/CT repeat motifs were dominant, accounting for 32.9% of all SSR; while the repeating motifs ACC/GGT, ATC/ATG, and AAG/CTT in the trinucleotides accounted for 10, 8.4 and 8.4%, respectively. The higher proportion of tetranucleotides corresponded to AAAAC/GTTTT, AAAAG/CTTTT, AAAAT/ATTTT and AAACC/GGTTT, while the remaining tetranucleotide, pentanucleotide and hexanucleotide repeating motifs were more dispersed, with low occurrence frequencies, and account only for 9.4% (Fig. 7).

## Discussion

Jerusalem artichoke is a hexaploid (2n = 6x = 102) outcrossing plant with a large genome [24], which utilizes tubers for asexual reproduction and has self-incompatibility, low hybridization rate, and a complex genetic structure. Compared with other large crops, bioinformatics resources on Jerusalem artichoke are relatively scarce. With the widespread use of transcriptome sequencing technology, mining for genetic resources is possible with low cost and high throughput. In this study, RNA-Seq technology was used to assemble and analyze the transcriptomes of different tissues at flowering stage, obtaining 63,089 unigenes. After analysis of the Nr and Swiss Prot protein databases in NCBI, 41,041 (65.05%) and 33,494 (53.09%) unigenes were not annotated as relevant information in each database, respectively. This phenomenon has been reported in the results of transcriptome sequencing in many species, such as *Abelmoschu sesculentus* [25], *Fraxinus velutina* [26], *Morus alba* L. [27], and other. This has been related to the short length of some Unigene fragments, the lack of information on the relevant database genes, and the possible presence of new genes in Jerusalem artichoke [28]. The GO database was used to analyze the functional classification and biological characteristics of the annotated genes. However, due to defects such as incomplete functional classification in the GO database, 41,738 unigenes have not been annotated to possible GO entries. Therefore, other bioinformatics methods are needed to supplement the functional classification of Jerusalem artichoke unigenes.

Through the analysis of amino acid sequence alignment, we found the unigenes related to fructan metabolism in Jerusalem artichoke had some sequence-conserved functional domains, all belonging to the 32 family of glycosyl hydrolase (Glycosylhydrolysase 32, GH-32). The phylogenetic tree was constructed to show fructosyltransferases (FBE) and vacuolar invertase constituted a branch of the Jerusalem artichoke phylogenetic tree, while extracellular hydrolase and cell wall transformase were clustered into one species, indicating FBE in plants may have evolved from vacuolar invertase [29, 30]. These similar evolutionary origins are supported by studies where synthetase 1-FFT has been observed to display degradative enzymatic function, while the degrading enzyme 1-FEH sometimes has partial synthesis ability [31, 32]. Further research is need to clarify the functions of currently known fructan metabolism-related enzymes. Indeed, although some genes involved in fructan metabolism in Jerusalem artichoke fructan have been cloned [32, 33], other genes in this metabolic pathway need further exploration.

Analysis of the expression of key unigenes in the synthesis and degradation of fructan in Jerusalem artichoke revealed 12 unigenes were expressed during tuber development. The metabolism of fructan is a complex processes. Fructan is a carbohydrate composed of multiple fructose groups linked by glycosidic bonds. Sucrose is a precursor of fructan synthesis, and different combinations of fructosyltransferases catalyze the synthesis of multiple types of fructans, without the involvement of phosphorylation or nucleotide cofactors [34]. Results indicate Unigene32177, which is related to fructan synthesis in Jerusalem artichoke, has similar expression patterns to sucrose invertases Unigene7092 and Unigene46471. Because the substrates are fructose and glucose produced by sucrose hydrolysis during inulin synthesis, fructan synthesis and sucrose conversion are thought to occur simultaneously. Current research on inulin-type fructan have focused on 1-SST, 1-FFT (two important genes involved in fructan synthesis) and FEH (which is ultimately responsible for the degradation of fructan). However, invertase INV is required for the complete degradation of fructan [35]. Because one the final product of FEH hydrolysis is sucrose, this reaction is inhibited by sucrose [36]. The outcomes of the present study indicate different INV have diverse expression levels in various stages of Jerusalem artichoke tuber development. It is hypothesized these functional differences among INV are expressed at a variety of stages, showing functional pleiotropy. More importantly, the synergistic effect of INV gene is necessary during fructose synthesis and degradation in Jerusalem artichoke tuber development. The sequencing data from the transcriptome of this species can provide a reliable data basis for the identification and assessment of the expression of the members of the INV gene family. Therefore, further study is required regarding the functional analysis of INV in fructose synthesis and degradation, and its involvement in signal transduction pathways.

There is currently no SSR marker available in Jerusalem artichoke, and only 6 pairs of SSR-labeled primers have been identified in the same sunflower of Jerusalem artichoke [34], which does not meet research needs. However, the direct use of Jerusalem artichoke genome information to develop SSR markers is an important prerequisite for systematic research on the inheritance and evolution of this species. In this study, the transcriptome sequence information of Jerusalem artichoke was used to conduct a SSR locus search, identifying 6635 SSR loci with a frequency of 10.52%, which was higher than 7.6% reported for *Cajanus cajan* L. [35], but lower than the 14.70% documented for tea [36]. Dinucleotides (45.24%) and trinucleotides (42.53%) appeared most frequently as repeat motifs in Jerusalem artichoke, which was consistent with most species [37]. The most frequent dinucleotide repeat motif was AG/CT, echoing studies in dicots such as *Hevea brasiliensis* [38] and *Vitis* L. [39]. The most abundant trinucleotide repeat was ACC/GGT, which is inconsistent with the previous reports where the main repeat type of trinucleotide in dicots was AAG/CTT [40].

## Conclusions

In this study, a database evaluation of the transcriptome of Jerusalem artichoke from the Qinghai Tibet Plateau was conducted with high throughput sequencing technology to obtain a great amount of data on transcripts and SSR loci, depicting the general expression characteristics of this species’ transcriptome, and identifying its key metabolism-related genes. Moreover, this study also provides a reference for subsequent investigation of the metabolism of fructan and its regulation in Jerusalem artichoke; along with abundant data for molecular biology research on Asteraceae Helianthus plants.

## Materials and methods

### Plant material and RNA extraction

The transcriptome data was derived from the results of high-throughput sequencing conducted with Illumina equipment in “Qingyu No. 1” Jerusalem artichoke in 2016. In March 2016, this variety was planted in the experimental site of the College of Agriculture and Forestry of Qinghai University (N36°43′45.21′′E101°44′57.69′′). After the roots (1 cm of thick root tip, 1 cm of young root tip), stems (1 cm at the base of the stem, 0.5 cm at the tip of the stem), leaves (0.1 g of the first euphylla, 0.1 g of the tender leaf), flowers (0.1 g of flower petals, 0.1 g of pollen) and tubers (0.1 g of the longitudinal epidermis, 0.1 g of the tuber center) were taken from samples in the flowering stage, RNA was extracted, and the mixed sample was formed in equal amount. Then poly (A) was added, and the sequencing linker was ligated to prepare a sequencing library for PCR amplification and subsequent sequencing.

### Illumina sequencing and de novo assembly

The PE125 sequencing method was used to construct the Jerusalem artichoke transcriptome library using the Illumina HiSeq™ 2500 sequencing platform. The original image data obtained by order were converted to raw reads via base recognition (base calling), and raw reads were filtered to get clean reads, followed by statistical analysis of the amount of raw data and its length. The original data was subjected to removal of sequencing joints, repeated redundant sequences, and low-quality sequence data. Then, the number of clean reads, their total length, Q20, Q30%, N%, and GC% were counted. Q20 represents the ratio of bases having a mass of not less than 20 after filtration, Q30 represents the ratio of bases having a mass of not less than 30 after filtration, and N represents the ratio of bases which were not determined after filtration. The assembly was performed using Trinity [41] software, the sequence was extended to contigs by overlap between sequences, and then transcripts were obtained by local assembly. Finally, Transcripts were homogenously clustered and spliced using TGICL [42] and Phrap software [43] to obtain a single gene cluster (unigene). Analytical projects included analysis of the sequencing assembly results, FPKM statistical analysis, and SSR analysis.

### Sequence annotation

By comparing the protein database of Jerusalem artichoke to the protein databases (E value <1e-5) with the Blastx alignment tool, functional annotation was performed according to the similarity of the genes, and the protein with the highest sequence similarity with the given unigene was selected, thus the relevant functional annotation information. All annotation details were merged to summarize the information retrieved in this process. The protein databases utilized include Nr (Non-redundant protein database), Swiss Prot (Swiss Prot protein database), KOG (Clusters of orthologous groups for eukaryotic complete genomes), Cluster), GO (Gene Ontology) and KEGG (Kyoto Encyclopedia of Genes and Genomes). In addition, gene annotation was performed in the latter to identify key genes involved in the biosynthetic pathway of fructan in Jerusalem artichoke.

### Expressed sequence tag-simple sequence repeat identification

Unigenes in the Jerusalem artichoke transcriptome data was investigated using microsatellite identification tool (MISA), Available online: http://pgrc.ipk-gatersleben.de/misa. The search criteria were: minimum repeat number of dinucleotide 6 times, trinucleotide 5 times, 4 nucleotides 4 times, 5 nucleotides 4 times, and hexanucleotide 4 times, and the SSR locus interval was set to a minimum of 100 bp. The text file generated was imported into Excel for basic statistical analysis. SSR occurrence frequency = number of searched SSR-containing unigene sequences / total number of unigene sequence; SSR distribution frequency = SSR number / total number of unigene sequences; average distance of SSR distribution = total unigene length / number of searched SSR.

### Quantitative polymerase chain reaction analysis

The test material was “Qingyu No.1”, the main cultivar of the Qinghai Tibet Plateau. Planting occurred in the middle of April 2016 in the experimental field of the Academy of agriculture and Forestry Sciences, Qinghai University, Xining, Qinghai. The normal cultivation method was implemented, with a row spacing of 1 m, and a plant spacing of 80 cm. Samples were taken during the expansion of the tubers, and a total of 4 samples were taken, until the plants withered. The tuber was set to repeat 3 times. Immediately after sampling, it was placed in liquid nitrogen for quick freezing, and then stored at − 80 °C in a refrigerator. RNA was extracted using the TIANGEN RNA prep Pure Plant Total RNA Extraction Kit (DP432), and then gelled with 1.2% agarose gel, as well as freshly prepared 0.5*TBE for 15 min with 150 V to assess RNA integrity. In addition, the TaKaRa reverse transcription kit PrimeScript™ RTMaster Mix (Perfect Real Time) (RR036B) was used to realize reverse transcription according to manufacturer’s instructions. The RNA sample amount was 1 μg, and the reaction system was 20 μL. Furthermore, the reverse-transcribed cDNA sample was then stored at − 20 °C for later use. We performed real-time qPCR on a 96-well reaction plate with Applied Biosystems SteponePlus using TaKaRa’s SYBR Premix Ex TaqTM (TliRNaseH Plus) kit (RR420B). The internal reference primer was selected from the 25S ribosomal RNA primer F of Jerusalem artichoke: CTGTCTACTATCCAGCGAAACCA, R: AGGGCTCCCACTTATCCTACAC. The total volume of the quantitative PCR reaction was 20 μL, containing 10 μL of SYBR Premix, 0.6 μL of the upstream and downstream primers, 3.4 μL of ddH2O, and 5 μL of 0.1* cDNA. The reaction conditions of qPCR were: Pre-denaturation at 95 °C for 30 s; 95 °C for 5 s, 60 °C for 30 s, 40 cycles. The fluorescence signal was collected during the extension phase at 60 °C to obtain the cyclic Ct threshold of different genes. At the end of the cycle, the dissolution curve was measured to evaluate the specificity of the PCR product. The dissolution curve program was 95 °C for 15 s, 60 °C for 1 min, and 95 °C for 15 s. Each sample contained 3 biological replicates during the quantification process, 3 replicates of the same sample, and 3 primer-free negative controls in each 96-well reaction plate. The relative expression results of the genes were calculated using 2^-ΔΔCT^ [44]. The HeatMap Tools at http://www.omicshare.com/tools/home/soft/heatmap/ were implemented to draw a heatmap with parameters set to: cluster_rows: TRUE, cluster_cols: TRUE, colors: green, black, red.

## Abbreviations

GO: Gene ontology
KEGG: Kyoto encyclopedia for genes and genomes
KOG: Clusters of orthologous groups for eukaryotic complete genomes
SSR: Simple sequence repeat

## References

[1] Saengkanuk A, Nuchadomrong S, Jogloy S, Patanothai A, Srijaranai S (2011). A simplified spectrophotometric method for the determination of inulin in Jerusalem artichoke (Helianthus tuberosus L.) tubers. Eur Food Res Technol.

[2] Wang Y-Z, Zou S-M, He M-L, Wang C-H (2015). Bioethanol production from the dry powder of Jerusalem artichoke tubers by recombinant Saccharomyces cerevisiae in simultaneous saccharification and fermentation. J Ind Microbiol Biotechnol.

[3] Lee Y-J, M-G L, Yu S-Y, Yoon W-B, Lee O-H (2014). Changes in physicochemical characteristics and antioxidant activities of Jerusalem artichoke tea infusions resulting from different production processes. Food Sci Biotechnol.

[4] Koutinas AA, Garcia IL, Kopsahelis N, Papanikolaou S, Webb C, Villar MA, López JA (2013). Production of fermentation feedstock from Jerusalem artichoke tubers and its potential for Polyhydroxybutyrate synthesis. Waste Biomass Valorization.

[5] Debez A, Belghith I, Friesen J, Montzka C, Elleuche S (2017). Facing the challenge of sustainable bioenergy production: could halophytes be part of the solution?. J Biol Eng.

[6] Phieler R, Voit A, Kothe E, Schippers A, Glombitza F, Sand W (2014). Microbially supported phytoremediation of heavy metal contaminated soils: strategies and applications. Geobiotechnology I: Metal-related Issues.

[7] Wardlaw CW, Reinders-Gouwentak CA, Champagnat P, Doorenbos J, Allsopp A, Allsopp A, Bloch R, Bonner JT, Bopp M, Brachet J, Brown R, Bünning E, Buvat R, Cantino EC, Champagnat P (1965). Organisation und Ausgestaltung des Sprosses. — organization and development of the shoot. Differentiation and Development / Differenzierung und Entwicklung: Part 1 / Teil 1.

[8] Apolinário AC, de Lima Damasceno BPG, de Macêdo Beltrão NE, Pessoa A, Converti A, da Silva JA (2014). Inulin-type fructans: a review on different aspects of biochemical and pharmaceutical technology. Carbohydr Polym.

[9] Barthomeuf C, Regerat F, Pourrat H (1991). High-yield ethanol production from Jerusalem artichoke tubers. World J Microbiol Biotechnol.

[10] Zhang M, Chen Q, Shen S (2011). Physiological responses of two Jerusalem artichoke cultivars to drought stress induced by polyethylene glycol. Acta Physiol Plant.

[11] Sharbatkhari M, Shobbar Z-S, Galeshi S, Nakhoda B (2016). Wheat stem reserves and salinity tolerance: molecular dissection of fructan biosynthesis and remobilization to grains. Planta.

[12] Fava F, Tuohy KM (2017). Inulin regulates endothelial function: a prebiotic smoking gun?. Nat Rev Gastroenterol Hepatol.

[13] Cruz-Cárdenas CI, Miranda-Ham ML, Castro-Concha LA, Ku-Cauich JR, Vergauwen R, Reijnders T, Van den Ende W, Escobedo-GraciaMedrano RM (2015). Fructans and other water soluble carbohydrates in vegetative organs and fruits of different Musa spp. accessions. Front Plant Sci.

[14] Aliasgharzadeh A, Khalili M, Mirtaheri E, Pourghassem Gargari B, Tavakoli F, Abbasalizad Farhangi M, Babaei H, Dehghan P (2015). A combination of prebiotic inulin and Oligofructose improve some of cardiovascular disease risk factors in women with type 2 diabetes: a randomized controlled clinical trial. Advanced Pharmaceutical Bulletin.

[15] Ritsema T, Smeekens S (2003). Fructans: beneficial for plants and humans. Curr Opin Plant Biol.

[16] Vijn I, Smeekens S (1999). Fructan: more than a reserve carbohydrate?. Plant Physiol.

[17] Edelman J, Jefford TG (1968). The mechanisim of fructosan metabolism in higher plants as exemplified in helianthus tuberosus. New Phytol.

[18] Higashiya H, Kobayashi K, Terada S. Fructan as a novel effective factor for mammalian cell. Culture. 2010:679–82.

[19] Shiomi N (1982). Purification and characterisation of 1F-fructosyltransferase from the roots of asparagus (asparagus officinalis L.). Carbohydr Res.

[20] Ueno K, Ishiguro Y, Yoshida M, Onodera S, Shiomi N (2011). Cloning and functional characterization of a fructan 1-exohydrolase (1-FEH) in edible burdock (Arctium lappa L.). Chem Cent J.

[21] Dassanayake M, Haas JS, Bohnert HJ, Cheeseman JM (2009). Shedding light on an extremophile lifestyle through transcriptomics. New Phytol.

[22] Lu T, Lu G, Fan D, Zhu C, Li W, Zhao Q, Feng Q, Zhao Y, Guo Y, Li W (2010). Function annotation of the rice transcriptome at single-nucleotide resolution by RNA-seq. Genome Res.

[23] Sangwan RS, Tripathi S, Singh J, Narnoliya LK, Sangwan NS (2013). De novo sequencing and assembly of Centella asiatica leaf transcriptome for mapping of structural, functional and regulatory genes with special reference to secondary metabolism. Gene.

[24] Bock DG, Kane NC, Ebert DP, Rieseberg LH (2014). Genome skimming reveals the origin of the Jerusalem artichoke tuber crop species: neither from Jerusalem nor an artichoke. New Phytol.

[25] Schafleitner R, Kumar S, C-y L, Hegde SG, Ebert A (2013). The okra (Abelmoschus esculentus) transcriptome as a source for gene sequence information and molecular markers for diversity analysis. Gene.

[26] Yan LP, Liu CL, Wu DJ, Li L, Shu J, Sun C, Xia Y, Zhao LJ (2016). De novo transcriptome analysis of Fraxinus velutina using Illumina platform and development of EST-SSR markers. Biol Plant.

[27] Liu CY, Liu XQ, Long DP, Cao BN, Xiang ZH, Zhao AC (2017). De novo assembly of mulberry (Morus alba L.) transcriptome and identification of candidate unigenes related to salt stress responses. Russ J Plant Physiol.

[28] Zhao X, Wang Q, Jiao Y, Huang R, Deng Y, Wang H, Du X (2012). Identification of genes potentially related to biomineralization and immunity by transcriptome analysis of pearl sac in pearl oyster Pinctada martensii. Mar Biotechnol.

[29] Kawakami A, Yoshida M (2005). Fructan:fructan 1-fructosyltransferase, a key enzyme for biosynthesis of graminan oligomers in hardened wheat. Planta.

[30] Stolze A, Wanke A, van Deenen N, Geyer R, Prufer D, Schulze Gronover C (2017). Development of rubber-enriched dandelion varieties by metabolic engineering of the inulin pathway. Plant Biotechnol J.

[31] Wim VE, Midori Y, Stefan C, Rudy V, Akira K (2005). Cloning, characterization and functional analysis of novel 6-kestose exohydrolases (6-KEHs) from wheat (Triticum aestivum). New Phytol.

[32] Xu H, Liang M, Xu L, Li H, Zhang X, Kang J, Zhao Q, Zhao H (2015). Cloning and functional characterization of two abiotic stress-responsive Jerusalem artichoke (Helianthus tuberosus) fructan 1-exohydrolases (1-FEHs). Plant Mol Biol.

[33] Van Der Meer IM, Koops AJ, Hakkert JC, Van Tunen AJ (1998). Cloning of the fructan biosynthesis pathway of Jerusalem artichoke. Plant J.

[34] Jung WY, Lee SS, Park HJ, Kim CW, Kwon S-Y, Jeon J-H, Kim H-S, Cho HS (2016). Comparative transcriptome profiling and SSR marker identification in three Jerusalem artichoke (Helianthus tuberosus L.) cultivars exhibiting phenotypic variation. Plant Biotechnol Rep.

[35] Dutta S, Kumawat G, Singh BP, Gupta DK, Singh S, Dogra V, Gaikwad K, Sharma TR, Raje RS, Bandhopadhya TK (2011). Development of genic-SSR markers by deep transcriptome sequencing in pigeonpea [Cajanus cajan (L.) Millspaugh]. BMC Plant Biol.

[36] Wu H, Chen D, Li J, Yu B, Qiao X, Huang H, He Y (2013). De novo characterization of leaf transcriptome using 454 sequencing and development of EST-SSR markers in tea (Camellia sinensis). Plant Mol Biol Report.

[37] Liang X, Chen X, Hong Y, Liu H, Zhou G, Li S, Guo B (2009). Utility of EST-derived SSR in cultivated peanut (Arachis hypogaea L.) and Arachiswild species. BMC Plant Biol.

[38] Feng SP, Li WG, Huang HS, Wang JY, Wu YT (2009). Development, characterization and cross-species/genera transferability of EST-SSR markers for rubber tree (Hevea brasiliensis). Mol Breed.

[39] Huang H, Lu J, Ren Z, Hunter W, Dowd SE, Dang P (2011). Mining and validating grape (Vitis L.) ESTs to develop EST-SSR markers for genotyping and mapping. Mol Breed.

[40] Morgante M, Hanafey M, Powell W (2002). Microsatellites are preferentially associated with nonrepetitive DNA in plant genomes. Nat Genet.

[41] Grabherr MG, Haas BJ, Yassour M, Levin JZ, Thompson DA, Amit I, Adiconis X, Fan L, Raychowdhury R, Zeng Q (2011). Full-length transcriptome assembly from RNA-Seq data without a reference genome. Nat Biotechnol.

[42] Pertea G, Huang X, Liang F, Antonescu V, Sultana R, Karamycheva S, Lee Y, White J, Cheung F, Parvizi B (2003). TIGR gene indices clustering tools (TGICL): a software system for fast clustering of large EST datasets. Bioinformatics.

[43] Ewing B, Hillier LD, Wendl MC, Green P, Ewing B, Hillier LD, Wendl MC, Green P (1998). Base-calling of automated sequencer traces UsingPhred. I. Accuracy assessment. Genome Res.

[44] Livak KJ, Schmittgen TD (2001). Analysis of relative gene expression data using real-time quantitative PCR and the 2−ΔΔCT method. Methods.

